# Application Progress of PALS in the Correlation of Structure and Properties for Graphene/Polymer Nanocomposites

**DOI:** 10.3390/nano12234161

**Published:** 2022-11-24

**Authors:** Xiaobing Han, Jie Gao, Tao Chen, Libing Qian, Houhua Xiong, Zhiyuan Chen

**Affiliations:** Hubei Key Laboratory of Radiation Chemistry and Functional Materials, School of Nuclear Technology and Chemistry & Biology, Hubei University of Science and Technology, Xianning 437100, China

**Keywords:** PALS, graphene, polymer, correlation, free volume, interfacial interaction, property

## Abstract

Giving a deep insight into the microstructure, and realizing the correlation between microstructure and properties is very important to the precise construction of high-performance graphene/polymer nanocomposites (GPN). For the promising application in microstructure characterization, much attention has been focused on the effective technique of positron annihilation lifetime spectroscopy (PALS). Based on the introduction of the basic principle, this review summarized the application progress of PALS in the correlation of microstructure and properties for GPN, especially for the characterization of free volume and interfacial interaction, and the correlation of these microstructures and properties.

## 1. Introduction

Due to the combination of the viscoelasticity of polymers and the functionality of nanofillers, polymer nanocomposites have been widely used in the field of composites. Owing to the excellent mechanical, electrical, thermal, and optical properties of graphene nanosheets, leading to the rapid development of graphene-based polymer nanocomposites [1,2,3,4,5]. It is well-known that the microstructure of polymer composites determines the performance of the prepared materials, therefore the microstructure characterization is highly desirable for the molecular design. For the GPN, the microstructure includes the interfacial interaction intensity (*β*) between graphene and polymer, the free volume (*V_f_*), and the free volume fraction (*f_v_*) of the composite. The *f_v_* and *β* are closely related to their transport properties, such as mechanical, electrical, thermal, and barrier properties. Therefore, the establishment of qualitative or even quantitative relationships between these microstructures and properties will be helpful for the precise construction of high-performance GPN [6,7,8,9,10].

Though many conventional and special techniques have been used for the structural characterization of GPN, due to various restrictions, it is very difficult to quantitatively reveal the microstructure such as *f_v_* and *β*. Consequently, it is unfavorable for the establishment of a relationship between these microstructures and properties [4,11]. Compared with other characterization techniques, PALS is the most sensitive method to detect the size and concentration of free volume in polymer composites [12,13,14,15]. With the development of PALS, this technology can not only characterize the free volume characteristics of polymer composites, but also can reveal the interfacial interaction between polymer and nanofiller [16,17,18,19], which provided a new approach for the relationship established between the microstructure and property.

Due to it being a powerful tool in microstructure characterization, PALS was widely used in the correlation of structure and properties for GPN in recent years. Though there are some reviews about the application of PALS in the structural characterization of polymer and corresponding composites [20,21,22,23], there are few reviews about the application of PALS in the structural characterization of GPN. To realize the precise construction of high-performance GPN, now is an appropriate time to summarize the application progress of PALS in the correlation of structure and properties for GPN. Firstly, the basic principle of PALS was introduced, including the correlation between the long-lived lifetime (*τ*_3_), intermediate lifetime intensity (*I*_2_), and properties, interfacial interaction, respectively. Secondly, the application of PALS in the structural characterization of graphene and graphene oxide was summarized. Finally, the application of PALS in the correlation of microstructure and properties for GPN was summarized, especially for the characterization of free volume and interfacial interaction, and the correlation of this microstructure and properties.

## 2. Application Principle of PALS in Polymer Composites Characterization

Since the discovery of positron by Anderson in 1932, the PALS technique was developed and used as a useful tool to determine the atomic scale defects for a wide variety of polymer and polymer composites [20,21]. Positrons generated by radioactive sources such as ^22^Na can diffuse into the polymer, annihilating directly with free electrons or indirectly by forming positronium (Ps). Both the generation and annihilation of positron will emit γ photons, the time interval (annihilation lifetime) of the emitted γ photons is highly dependent on the free volume and interface interaction. The Ps exist in two spin states, namely *para*-positronium (p-Ps) and *ortho*-positronium (o-Ps). Generally, four annihilation lifetime components can be observed for crystalline polymers (*τ*_1_, *τ*_2_, *τ*_3_, *τ*_4_), the *τ*_1_ and *τ*_2_ were considered to be the lifetime of p-Ps self-annihilation and positron direct annihilation, and the *τ*_3_ and *τ*_4_ were considered to the pick-off annihilation lifetime for o-Ps in amorphous and crystalline region. As almost no crystalline region exists in the amorphous polymer, only three annihilation lifetime components can be observed [22,23].

At present, the application of PALS in structure characterization for polymer composites was mainly focused on the measurement of annihilation lifetime [24,25,26,27,28]. On the one hand, the *V_f_* and *f_v_* can be calculated with the value of *τ_3_*, and the relationship can be established between the obtained *f_v_* and the material properties [29,30,31,32,33]. On the other hand, the *β* can be calculated with the value of *I*_2_, which can give a deep insight into the dispersion state of filler and corresponding properties [16,17,18,19].

### 2.1. Correlation between f_v_ and Properties Based on τ_3_

The most important application of PALS in structure characterization for polymer and polymer composites is the probing of *V_f_*, which is closely related to the glass transition of polymer. A simple quantum mechanical model was proposed in 1972 [34], Ps is considered to be localized in a spherical infinite potential of radius *R* with a surface electron layer of thickness ΔR (the empirical value is 0.1656 nm, which has been determined with materials having well know free volume hole size), and the pick-off annihilation lifetime for o-Ps (*τ*_3_) is correlated with R of the *V_f_*. The correlation of *τ*_3_ and R is as follows (Equation (1)) [21]:

(1) $$\tau_{3}=\frac{1}{2}{ [1-\frac{R}{R+\Delta R}+\frac{1}{2\pi }\sin\left(\frac{2\pi R}{R+\Delta R}\right)]}^{-1}$$

According to the above-mentioned model, the volume of holes in the polymeric materials can be calculated through Equation (2) [21]:

(2) $$V_{f}=\frac{4}{3}\pi R^{3}$$

The formation probability of o-Ps (*I*_3_) is correlated with the intensity of the free volume, and the free volume fraction (*f_v_*) of a polymeric material is calculated with Equation (3). *A* is a proportionality constant, which has been estimated to be 0.0018 nm^−3^ to a large number of polymers [21]. For convenience, relative free volume fraction (*f_r_*) (Equation (4)) was defined and used.

(3) $$f_{v}={AV}_{f}I_{3}$$

(4) $$f_{r}=V_{f}I_{3}$$

The PALS was widely used for the *V_f_*, *f_v,_* and corresponding property investigation for a large number of polymer composites. As reported by Wang and co-workers [26], the glass transition temperature (*T_g_*) and viscoelastic properties of polycarbonate/multi-walled carbon nanotube (PC/MWCNT) composites can be revealed by PALS. The higher the MWCNT content, the lower the *T_g_*, which can be ascribed to the large *V_f_* and the enhanced polymer mobility. Based on the free volume theory, a direct linear correlation between *f_v_* and the viscoelastic property of PC/MWCNT has been obtained using the WLF equation. In addition, based on the results of *V_f_* and *f_v_* obtained from PALS, the property change rule of many polymer composites can be explained [24,25,26,27,28,29,30,31,32,33]. With the deepening of the investigation, a quantitative relationship between the *V_f_*, *f_r_*, and properties was established. In the conductivity investigation of poly(ether urethane)-LiClO_4_ composites, a direct relationship between the conductivity (*σ*) and *f_r_* has been established based on the experimental measurements (Equation (5)) [27]. In the gas barrier investigation of styrene-butadiene rubber composites, the gas barrier is mainly influenced by *f_v_* and tortuous diffusional path effects, and a direct relationship between the diffusion coefficient (*D*) and *f_v_* has also been established (Equation (6)) [28].

(5) $$\log_{10}\sigma \left(T\right)=\log_{10}\sigma \left(T_{g}\right)+C_{1}\left(1-\frac{f_{g}}{f_{r}}\right)$$

(6) $$D=\frac{A}{Τ}\exp\left(-B/fv\right)$$

### 2.2. Investigation of β Based on I_2_

The intermediate lifetime *τ*_2_ is assigned to the annihilation of positrons trapped in various vacancies, and the variation of the intermediate lifetime intensity (*I*_2_) can be used to characterize the interfacial interaction between polymer and nanofiller in the polymer composites [16,17]. According to the simple mixture rule, if no interfacial interaction exists between polymer and nanofiller, the *I*_2_ only comes from the annihilation in the polymer matrix and nanofiller. Under this condition, *I*_2_ is linearly correlated with the content of the nanofiller (Equation (7)).

(7) $$I_{2}=I_{2}^{N}W+I_{2}^{P}\left(1-W\right)$$

Actually, the interfacial interaction always exists in polymer composites, thus deviations occurred between the theoretical results and the experimental results [16,17]. According to the deviation of theoretical value and experimental value of *I*_2_, the interfacial interaction intensity and the dispersion state of the filler can be qualitatively evaluated. In the interfacial interaction investigation of phenol-formaldehyde resin/carbon nanotube composite [35], the experimental results of *I*_2_ show negative deviation indicating weak interfacial interactions between resin and nanotubes. In the interfacial interaction investigation of epoxy resin/modified clay composites [36], the deviation from the expected intensity value is maximum for the 1 wt.% clay sample, indicating the strongest interaction for the sample containing 1 wt.% clay. However, with the linear correlation Equation (7), the *β* cannot be investigated in quantitative.

The interaction parameter *β* was introduced to characterize the interfacial interaction intensity between polymer and filler, which can be calculated according to Equation (8) [18], where the superscripts *C*, *N*, and *P* refer to composite, nanofiller, and polymer, and *W* is the weight ratio of nanofiller.

(8) $$I_{2}^{C}=I_{2}^{N}W+I_{2}^{P}\left(1-W\right)+\beta I_{2}^{N}WI_{2}^{P}\left(1-W\right)$$

In the investigation of interfacial interaction and structural transition for epoxy/nanotube composites by PALS [19], the *β* of the composites with different nanotube content was calculated through Equation (8). The results revealed that stronger interfacial interactions exist in the composites with modified nanotubes, which is in good agreement with the determination of *T_g_*.

## 3. Application of PALS in Graphene and Graphene Oxide Characterization

As the PALS investigation of GPN involves the positron annihilation parameters of graphene and graphene oxide (GO), thus the application of PALS in these fillers’ characterization has also been summarized. As so far, there are no reports about the PALS investigation for pristine graphene, which is obtained with liquid exfoliation or chemical vapor deposition and possesses a perfect *sp*^2^ hybrid structure. The positron annihilation parameters of graphene used in the calculation were obtained from reduced graphene oxide (RGO), which was obtained with chemical or thermal reduction of GO and partially restoring the π network.

The PALS parameters of RGO and GO are shown in Table 1. As shown in the table, there is a big difference between the annihilation parameters of the fillers prepared with different methods. Chakarabarti and co-workers investigated the PALS of RGO obtained with the reduction of polyaniline [37], in fact, the obtained RGO contained an impurity of polyaniline. The intermediate lifetime intensity (*I*_2_) of this RGO is 63.8%, which has been widely used in other groups’ investigations for the calculation of interfacial interaction intensity (*β*). The PALS investigation of RGO obtained with potassium carbonate was conducted by Peng and co-workers [38], single-layered RGO with high purity was produced, and the value of *I*_2_ was as high as 94.78%. There is no obvious difference in the annihilation lifetime for GO obtained with traditional Hummers methods [39] and modified Hummers methods [40], but there is a big difference in their intensity, which demonstrates that the preparation methods have a great impact on the concentration of defects or holes.

## 4. Application of PALS in Structure-Property Correlation for GPN

Due to it being a powerful tool in microstructure characterization, PALS was widely used in the investigation for GPN in recent years [41], including the GPN used for nanofiltration membranes [42,43], gas barrier [44,45], gas separation [46,47], fuel cells [48], supercapacitors [49]. The correlation of structure and property for GPN is also focused on the *f_v_* and *β*, which can be obtained with the determination of *τ*_3_ and *I*_2_.

**Figure 1:**
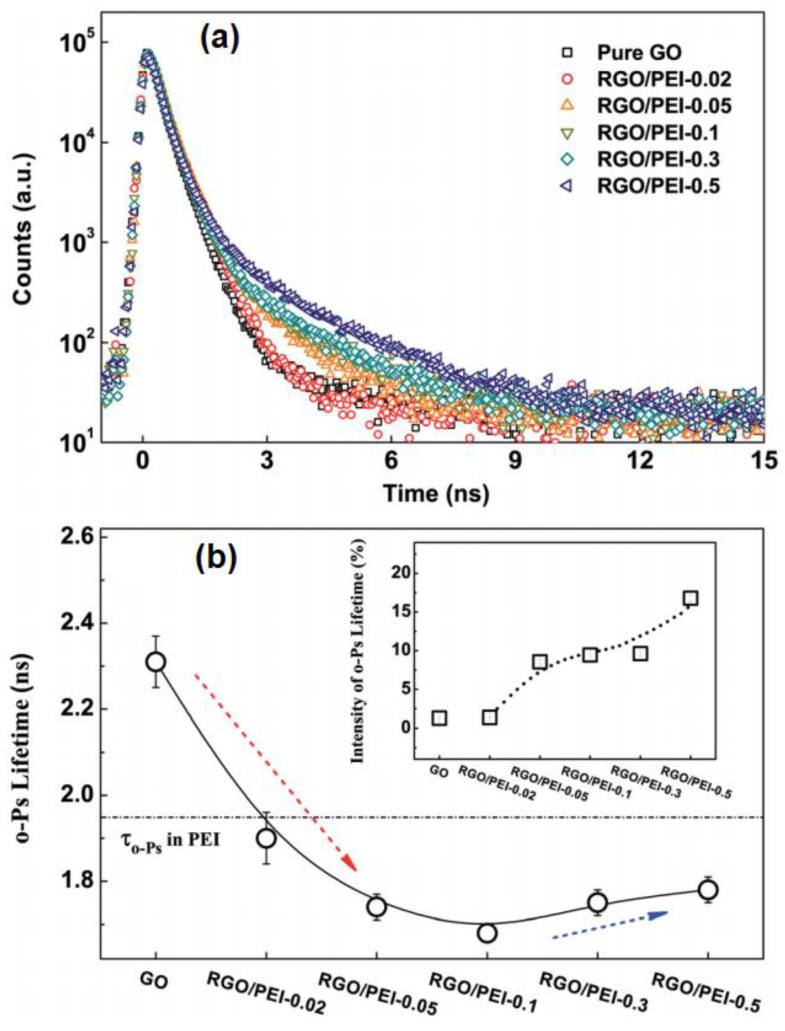
(**a**) The raw PALS spectra of PEI/RGO; (**b**) the variations in o-Ps lifetime and the intensity in the PEI/RGO composite film [44].

In the correlation between gas permeation and microstructure of RGO/PEI composite films, PALS was used to reveal the free volume (Figure 1) [44]. The raw PALS spectra for all samples are shown in Figure 1a, obvious differences in the decay behavior can be observed for different samples. The analysis program of PASA and LT was applied to analyze the raw PALS spectra, the results revealed that only one long-lived o-Ps lifetime can be derived from the spectra for all samples, and the *τ*_o-Ps_ and *I*_o-Ps_ are displayed in Figure 1b.

### 4.1. Correlation of Free Volume Fraction and Property

The *V_f_* and *f_v_* of GPN are closely related to their transport properties, such as phase change temperature, conductivity, gas barrier, thermal conductivity, etc. A quantitative relationship was established for GPN based on the *f_v_*, according to different physical models.

#### 4.1.1. Correlation between *f_v_* and Phase Change Temperature

**Figure 2:**
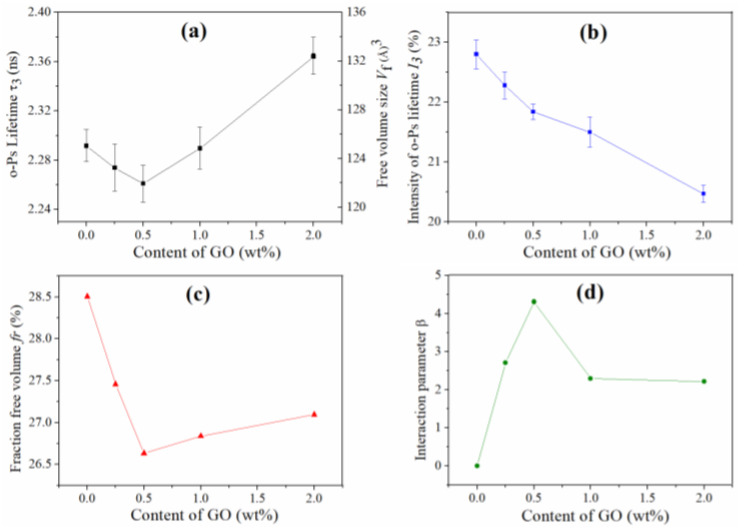
(**a**) o-Ps lifetime τ_3_ and free volume size *V_f_*, (**b**) o-Ps lifetime intensity *I*_3_, (**c**) fractional free volume *f*_r_, (**d**) interfacial interaction *β* of the GO/WPU composites [51].

Free volume is an important characteristic of polymer and their composites. Higher *f_v_* can provide more movement space for polymer segments in composites, which is beneficial for the movement of the polymer chain, leading to the decrease of glass transition temperature (*T_g_*) or melting point (*T_m_*) [50]. In the PALS investigation of GO/polyurethane composites (Figure 2) [51], the effect of GO content on the *f_r_* was studied. With the increase of GO content, the *f_r_* decreased at low GO content and increased at high GO content, which can be ascribed to the different dispersion states and interfacial interactions. The changing trend of *T_g_* is contrary to *f_r_*, lowest *f_r_* was obtained for the composites containing 0.5 wt.% GO, and the highest *T_g_* was observed for these composites. In another work of this group, the effect of graphene content on the *f_r_* for graphene/polyethylene composites was investigated [52]. With the increase of graphene content, the *f_r_* of the composites decreased gradually, leading to the increase of the *T_m_*.

#### 4.1.2. Correlation between *f_v_* and Conductivity

The *f_v_* of polymer composites is also closely related to conductivity. Lower *f_v_* can promote the formation of interfacial areas with high density for GPN, limiting the movement and relaxation of polymer segments. This facilitates the formation of conductive networks, leading to the conductivity improvement of GPN [53,54,55,56]. In the PALS investigation of the γ irradiation effect on the conductivity of polyvinyl alcohol/polyethylene glycol/graphene (PVA/PEG/RGO) composites, the correlation between conductivity and *V_f_* was established (Equation (9)) [57]. Through the data fitting of conductivity (*σ*) and *V_f_*, the critical free volume V_i_* was obtained. For the nonirradiated and irradiated PVA/PEG/RGO samples, the V_i_* for the charge carriers/ion transport is 80.34 and 82.16 Å^3^ respectively. In the investigation of polycarbonate (PC)/RGO composites, the conductivity and *f_r_* were correlated with PALS (Equation (10)) [58]. The value of *f*_0_, *f_d_*, and *b* was calculated to be 25.62%, 0.5663%, and 1.047 × 10^−6^ S/m, respectively.

(9) $$\sigma =\sigma_{0}\exp(-\frac{\gamma V_{i^{*}}}{V_{f}})$$

(10) $$f_{r}=f_{0}+f_{d}e^{-\frac{\sigma }{b}}\;$$

#### 4.1.3. Correlation between *f_v_* and Gas Barrier

The *f_v_* of polymer composites is also closely related to the permeability of gas and water. Lower *f_v_* can promote the formation of a zigzag channel effect in the GPN, which can decrease the transport rate of small molecules, leading to the enhancement of the barrier properties [59,60,61,62]. In the investigation of the alcohol dehydration performance of alginate composite with RGO and graphene quantum dots [63], the co-dopping membrane has the better alcohol-blocking capability. Because the diameter of free volume for the co-dopping membrane is 3.109 Å, which is smaller than the kinetic diameter of methanol (3.8 Å) but large enough for the transport of water (2.6 Å), leading to better performance for methanol/water separation. In the investigation of the orientation effect of sulfonated GO (SG) on the gas barrier for SG/nafion composite membrane, the *f_v_* under different orientations was determined with PALS [30]. In addition, the diffusion coefficient (*D_c_*) can be calculated with Equation (11), thus the orientation effect of SG on the gas barrier can be revealed with PALS.

(11) $$D_{c}=Ae^{\left(-B/fv\right)}$$

#### 4.1.4. Correlation between *f_v_* and Thermal Conductivity

The *f_v_* of polymer composites is also closely related to thermal conductivity. This can be ascribed to that the free volume holes own a radius at the angstrom level and can be an effective center for phonon scattering, while the phonon is very important for the transportation of heat. Thus, the larger the *f_v_*, the lower the thermal conductivity [64]. In the investigation of RGO content on the thermal conductivity of porous PVA/RGO composites [64], the correlation between *f_v_* and thermal conductivity was established (Equation (12)). Increasing free volume holes of PVA/RGO composites lead to an enhancement in the phonon scattering and hence a decrease in the thermal conductivity.

(12) $$\kappa =\kappa_{m}\exp\left(-\frac{fv}{t}\right)+\kappa_{0}$$

### 4.2. Correlation of Interfacial Interaction Intensity and Property

The interfacial interaction between polymer and filler is not only closely related to the dispersion state of graphene, but also closely related to the composite properties, including mechanical strength, conductivity, thermal conductivity, and gas barrier. According to the relationship between intermediate lifetime intensity (*I*_2_) and interfacial interaction intensity (*β*) (Equation (8)), the correlation between interfacial interaction and property was established.

#### 4.2.1. Correlation between *β* and Mechanical Property

**Figure 3:**
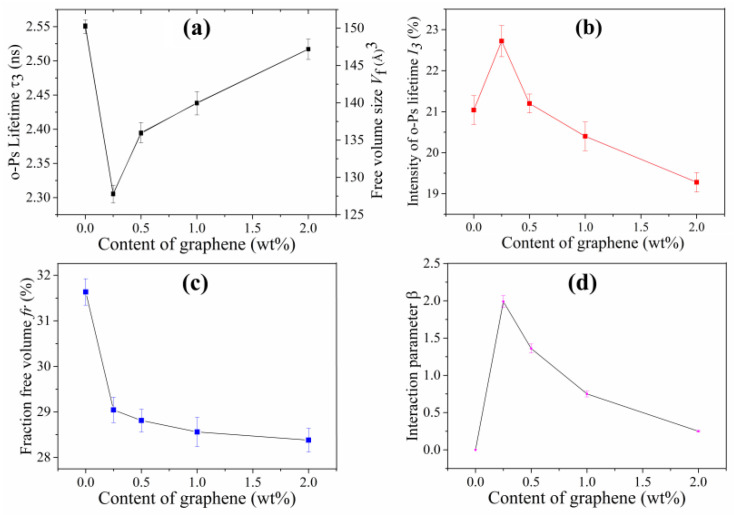
(**a**) Long lifetime (*τ*_3_)and free volume size (*V_f_*), (**b**) Long lifetime intensity (*I_3_*), (**c**) relative free volume fraction (*f_r_*), (**d**) interaction parameter (*β*) of the graphene/PE composites [52].

In the investigation of GO/polyurethane composites [51], the effect of GO content on the *β* was revealed with PALS through Equation (8), and a correlation between the *β* and mechanical property was established. The change of the composite tensile strength is consistent with the variation of *β*, because the tensile strength is closely related to the interfacial interaction, which can transfer the stress from the soft polymer matrix to the hard nanofiller. A similar phenomenon has also been observed in graphene/polyethylene composites (Figure 3) [52], which demonstrated that the relationship between interfacial interaction and mechanical property can be established with the PALS technique.

#### 4.2.2. Correlation between *β* and Conductivity

In the investigation of PC/RGO composites [58], the change of *β* as a function of RGO content was revealed. The composite with 0.5 wt.% RGO has the largest *β*, suggesting the interfacial interaction is strongest when the conductive network is formed. High *β* is beneficial for the formation of high-density interfacial regions, which can limit the movement and relaxation of the polymer chain, inducing the formation of conductive networks [53,54,55,56].

#### 4.2.3. Correlation between *β* and Thermal Conductivity

The interfacial interaction between polymer matrix and filler has an important effect on thermal conductivity, which dominates the delivery of phonon between filler and polymer [62]. In the investigation of the PVA/RGO composite [64], the *β* between PVA and RGO was also revealed through Equation (8). The interfacial thermal resistance increased with the increase of *β*, and reached the highest at the load level of 0.5 wt.%. This can be attributed to the high specific surface area can provide more functional sites, which can scatter phonons and dampen the phonons’ vibration amplitude at the surface, leading to higher thermal resistance.

#### 4.2.4. Correlation between Interfacial Interaction and Gas Barrier

Although the *β* was not revealed in the gas barrier investigation for GPN till now, yet the correlation between interfacial interaction and the gas barrier has been established in qualitative. Strong interfacial interaction between the polymer matrix and GO/RGO can limit the motion of the polymer chain, consequently, reducing the *f_v_* and increase the tortuous path, enhancing the gas barrier properties [44,59,60,61,62].

Except for the PALS, there is another positron annihilation technique namely Doppler broadening spectra (DBS) of annihilation radiation, which has also been widely used in the microstructure characterization for GPN in recent years [42,43]. The DBS can probe the energy broadening from electron motion in the atoms or molecules at the site where the annihilation takes place, which represents the momentum density in the longitudinal direction of annihilation radiation. The parameterized value (*S* parameter) in the low momentum region of DBS indicates the extent of free volumes in the substrates of polymeric systems [22].

The positron lifetime in polymer composites is sensitive to the size of free volume, while the momentum distribution of electrons in DBS is more sensitive to their chemical environments [65,66]. In the investigation of polyzwitterion membrane via assembly of GO-based core-brush nanosheet [67], slow positron annihilation DBS was used to reveal the microstructure, the smaller *S* parameter indicates a more compact membrane structure. In the investigation of spirobisindane-functionalized graphene oxide (SFGO)/ polyimide nanocomposite membranes [68], the *S* parameter as a function of positron incident energy was provided. As the author stated, the larger of the *S* parameter, the larger the free volume. Obvious drops were observed at energy less than 0.8 KeV, these drops occurred when positrons are closer to the surface, and the defect and different chemical nature of the surface can be detected. In the investigation of GO-modified Poly(N-isopropylacrylamide) (mPNIPAm) membranes [69], DBS was used to qualitatively yield information about the free volume. A minimum *S* parameter of 0.456 was observed in the deposition layer of GO, and the *S* parameter of the deposition layers after the GO grafted to mPNIPAm in the order of GO-PN_COOH,NH2_ (0.471) > GO-PN_COOH_ (0.461) > GO-PN_CONH2_ (0.459).

## 5. Conclusions and Future Prospects

Due to their excellent physical and chemical properties, graphene-based polymer nanocomposites have been widely studied and applied. It has become an important investigation direction of the precise construction of high-performance GPN, which is highly desirable for the revealing of the microstructure that determines transport properties, including *f_v_*, *β*, and the correlation of this microstructure and property. The PALS technique can not only reveal the free volume characteristic of GPN, but also be used to investigate the interfacial interaction between graphene and polymer matrix, which provided a new approach for the construction of the relationship between the structure and property of such composite. Combined with the condensed state physical model of materials, the quantitative relationship between the *f_v_* and conductivity, thermal conductivity, and barrier performance of this composite can be established, which lays the foundation for the precise construction of GPN. With the development of high-performance GPN, the quantitative relationship between *f_v_* and more properties will be established. The interfacial interaction determines the dispersion state of graphene, which is also closely related to the properties of the GPN. Though the *β* parameter can be calculated now, yet there are no reports about the quantitative correlation between *β* and properties, which need more attention. In addition, the *S* parameter originating from DBS is another important parameter for the microchemical environment investigation. However, there are few reports about the quantitative correlation between the *S* parameter and properties, which also need to be paid more attention.

In fact, except for the GPN, PALS has been widely used in the investigation of other materials, such as new synthetic polymers, novel inorganic compound, and their composites. On the other hand, except for the PALS, other new positron annihilation techniques such as Doppler broadening spectra (DBS), and angular momentum correlation (AMC) can also be used for the microstructure characterization of materials. With the deepening of research, a more quantitative relationship will be established for more materials according to the different physical model, which will promote the precise construction of new materials.

## Figures and Tables

**Table 1:** PALS parameters of RGO and GO.

Sample	*τ*_1_(ps)/*I*_1_(%)	*τ*_2_(ps)/*I*_2_(%)	*τ*_3_(ps)/*I*_3_(%)	Reference
RGO	63/36	402/63.8	4400/0.2	[37]
RGO	193.2/5.22	337.6/94.78	-	[38]
GO	197/21.39	388/78.44	2190/0.17	[39]
GO	200/54.2	440/42.0	2420/3.8	[40]

## Data Availability

Not applicable.

## References

[1] Kinloch I.A., Suhr J., Lou J., Young R.J., Ajayan P.M. (2018). Composites with carbon nanotubes and graphene: An outlook. Science.

[2] Yu X.W., Cheng H.H., Zhang M., Zhao Y., Qu L.T., Shi G.Q. (2017). Graphene-based smart materials. Nat. Rev. Mater..

[3] Huang X., Qi X.Y., Boey F., Zhang H. (2012). Graphene-based composites. Chem. Soc. Rev..

[4] Han X.B., Kong H., Chen T., Gao J., Zhao Y., Sang Y.N., Hu G.W. (2021). Effect of π–π stacking interfacial interaction on the properties of graphene/poly(styrene-*b*-isoprene-*b*-styrene) composites. Nanomaterials.

[5] Sang Y.N., Miao P.P., Chen T., Zhao Y., Chen L.F., Tian Y.Y., Han X.B., Gao J. (2022). Fabrication and evaluation of graphene oxide/hydroxypropyl cellulose/chitosan hybrid aerogel for 5-fluorouracil release. Gel.

[6] Han X.B., Gao J., Chen T., Zhao Y. (2020). Interfacial interaction and steric repulsion in polymer-assisted liquid exfoliation to produce high-quality graphene. Chem. Pap..

[7] Han X.B., Gao J., Hu G.W., Tang X.Q., Chen T. (2020). Effect of hydrocarbon polymer, feed ratio, and interfacial interaction on the liquid exfoliation of graphite. J. Nanopart. Res..

[8] Yao Y.J., Gao J., Bao F., Jiang S.F., Zhang X., Ma R. (2015). Covalent functionalization of graphene with polythiophene through suzuki coupling reaction. RSC Adv..

[9] Gao J., Bao F., Zhu Q.D., Tan Z.F., Chen T., Cai H.H., Zhao C., Cheng Q.X., Yang Y.D., Ma R. (2013). Attaching hexylbenzene and poly(9,9-dihexylfluorene) to brominated graphene via Suzuki coupling reaction. Polym. Chem..

[10] Gao J., Bao F., Feng L.L., Shen K.Y., Zhu Q.D., Wang D.F., Chen T., Ma R., Yan C.J. (2011). Functionalized graphene oxide modified polysebacic anhydride as drug carrier for levofloxacin controlled release. RSC Adv..

[11] Huang X., Yin Z., Wu S., Qi X., He Q., Zhang Q., Yan Q., Boey F., Zhang H. (2011). Graphene-based materials: Synthesis, characterization, properties, and applications. Small.

[12] Yang Q.G., Olsson P. (2022). Identification and evolution of ultrafine precipitates in Fe-Cu alloys by first-principles modelling of positron annihilation. Acta Mater..

[13] Olguin G., Yacou C., Motuzas J., Butterling M., Meulenberg W.A., Smart S., Costa J.C. (2022). Surfactant functionalised cobalt silica membranes-Gas permeation and thin film positron annihilation lifetime spectroscopy characterisation. J. Membr. Sci..

[14] Wang Z.H., Dong X.M., Chen Z.Y., Xiong H.H., Gao J., Du J.F., Tang X.Q., Zhang Q.K., Qian L.B., Chen Z.Q. (2021). Dependence of the ferromagnetism on vacancy defect in annealed In_2_O_3_ Nanocrystals: A positron annihilation study. Phys. Status Solidi A.

[15] Liu Q., Liu J.J., Li M.D., Yu T., Hu M.M., Jia P.Y., Qi N., Chen Z.Q. (2022). Plasticization of a novel polysulfone based mixed matrix membrane with high-performance CO_2_ separation studied by positron annihilation. Colloid Surface A.

[16] Wang B., Qi N., Gong W., Li X.W., Zhen Y.P. (2007). Study on the microstructure and mechanical properties for epoxy resin/montmorillonite nanocomposites by positron. Radiat. Phys. Chem..

[17] Zeng M.F., Sun X.D., Xiao H.Q., Ji G.Z., Jiang X.W., Wang B.Y., Qi C.Z. (2008). Investigation of free volume and the interfacial, and toughening behavior for epoxy resin/rubber composites by positron annihilation. Radiat. Phys. Chem..

[18] Liu J., Jean Y.C. (1995). Free-volume hole properties of polymer blends probed by positron annihilation spectroscopy: Miscibility. Macromolecules.

[19] Zhou W., Wang J.J., Gong Z.L., Gong J., Qi N., Wang B. (2009). Investigation of interfacial interaction and structural transition for epoxy/nanotube composites by positron annihilation lifetime spectroscopy. Appl. Phys. Lett..

[20] Shantarovich V.P. (2022). Positron annihilation and permeation of amorphous polymers. J. Membr. Sci. Res..

[21] Sharma S.K., Pujari P.K. (2017). Role of free volume characteristics of polymer matrix in bulk physical properties of polymer nanocomposites: A review of positron annihilation lifetime studies. Prog. Polym. Sci..

[22] Jean Y.C., Horn J.D., Hung W.S., Lee K.R. (2013). Perspective of positron annihilation spectroscopy in polymers. Macromolecules.

[23] Pethrick R.A. (1997). Positron annihilation-A probe for nanoscale voids and free volume. Prog. Polym. Sci..

[24] Zhao Y.L., Huang R.J., Wu Z.X., Zhang H., Zhou Z.R., Li L.F., Dong Y., Luo M., Ye B.J., Zhang H.J. (2021). Effect of free volume on cryogenic mechanical properties of epoxy resin reinforced by hyperbranched polymers. Mater. Des..

[25] Xia R., Cao X.Z., Gao M.Z., Zhang P., Zeng M.F., Wang B.Y., Wei L. (2017). Probing sub-nano level molecular packing and correlated positron annihilation characteristics of ionic cross-linked chitosan membranes using positron annihilation spectroscopy. Phys. Chem. Chem. Phys..

[26] Yan X.L., Gong Z.L., Gong J., Gao S., Wang B., Ruan X.F. (2013). Investigation of the glass transition and viscoelastic properties of polycarbonate/multi-walled carbon nanotube composites by positron annihilation lifetime spectroscopy. Polymer.

[27] Wang B., Li S.Q., Wang S.J. (1997). Correlation between the segmental motion and ionic conductivity of poly(ether urethane)-LiClO4 complex studied by positron spectroscopy. Phys. Rev. B.

[28] Wang Z.F., Wang B., Qi N., Zhang H.F., Zhang L.Q. (2005). Influence of fillers on free volume and gas barrier properties in styrene-butadiene rubber studied by positrons. Polymer.

[29] Wan J.M., Nian M.J., Yang C., Ge K., Liu J.J., Chen Z.Q., Duan J.G., Jin W.Q. (2022). Interface regulation of mixed matrix membranes by ultrathin MOF nanosheet for faster CO_2_ transfer. J. Membr. Sci..

[30] Yin C.S., He C.Q., Liu Q.C., Xiong B.Y., Li J.J., Zhou Y.W. (2021). Effect of the orientation of sulfonated graphene oxide (SG) on the gas-barrier properties and proton conductivity of a SG/Nafion composite membrane. J. Membr. Sci..

[31] Wang Z., Qian L.B., Peng X.Y., Huang Z., Yang Y., He C.Q., Fang P.F. (2021). New aspects of degradation in silicone rubber under UVA and UVB irradiation: A gas chromatography-mass spectrometry study. Polymers.

[32] Liu Q., Xu M.D., Zhao J., Wang Y.D., Qi C.Z., Zeng M.F., Xia R., Cao X.Z., Wang B.Y. (2018). Insightful understanding of the correlations of the microstructure and catalytic performances of Pd@chitosan membrane catalysts studied by positron annihilation spectroscopy. RSC Adv..

[33] Yang L., Yang L.Q., Ma K., Wang Y., Song T., Gong L.L., Sun J., Zhao L., Yang Z.H., Xu J.M. (2021). Free volume dependence of dielectric behaviour in sandwich-structured high dielectric performances of poly(vinylidene fluoride) composite films. Nanoscale.

[34] Tao S.J. (1972). Positronium annihilation in molecular substances. J. Chem. Phys..

[35] Sharma S.K., Prakash J., Sudarshan K., Maheshwari P., Sathiyamoorthy D., Pujari P.K. (2012). Effect of interfacial interaction on free volumes in phenol formaldehyde resin-carbon nanotube composites: Positron annihilation lifetime and age momentum correlation studies. Phys. Chem. Chem. Phys..

[36] Patil P.N., Sudarshan K., Sharma S.K., Maheshwari P., Rath S.K., Patr M., Pujari P.K. (2012). Investigation of nanoscopic free volume and interfacial interaction in an epoxy resin/modified clay nanocomposite using positron annihilation spectroscopy. ChemPhysChem.

[37] Rana U., Nambissan P.M., Malik S., Chakarabarti K. (2014). Effects of process parameters on the defects in graphene oxide-polyaniline composites investigated by positron annihilation spectroscopy. Phys. Chem. Chem. Phys..

[38] He D.N., Peng Z., Gong W., Luo Y.Y., Zhao P.F., Kong L.X. (2015). Mechanism of a green graphene oxide reduction with reusable potassium carbonate. RSC Adv..

[39] Gong W., He D.N., Tao J.L., Zhao P.F., Kong L.X., Luo Y.Y., Peng Z., Wang H. (2015). Formation of defects in the graphite oxidization process: A positron study]. RSC Adv..

[40] Panzaradsa G., Consolati G., Scavi M., Longhi M., Quasso F. (2019). Convenient preparation of graphene oxide from expandable graphite and its characterization by positron annihilation lifetime spectroscopy. C-J. Carbon Res..

[41] Gorelov B.M., Mischanchuk O.V., Sigareva N.V., Shulga S.V., Gorb A.M., Polovina O.I., Yukhymchuk V.O. (2021). Structural and dipole-relaxation processes in epoxy-multilayer graphene composites with low filler content. Polymers.

[42] Shen Q., Lin Y.Q., Zhang P.F., Segawa J., Jia Y.D., Istirokhatun T., Cao X.Z., Guan K.C., Matsuyama H. (2021). Development of ultrathin polyamide nanofilm with enhanced inner-pore interconnectivity via graphene quantum dots-assembly intercalation for high-performance organic solvent nanofiltration. J. Membr. Sci..

[43] Lin Y.Q., Shen Q., Kawabata Y., Segawa J., Cao X.Z., Guan K.C., Istirokhatun T., Yoshioka T., Matsuyama H. (2021). Graphene quantum dots (GQDs)-assembled membranes with intrinsic functionalized nanochannels for high-performance nanofiltration. Chem. Eng. J..

[44] Yin C.S., Du X., Ding Z., Zeng Q., Li X., He C.Q., Xiong B.Y., Li J.J., Zhou Y.W. (2022). Gas permeation and microstructure of reduced graphene oxide/polyethyleneimine multilayer fifilms created via recast and layer-by-layer deposition processes. RSC Adv..

[45] Liu G.J., Yang F., Liu W.B., Bai Y.J., Han C., Jiao W.C., Wang P.P., Wang R.G. (2021). Ultra-high gas barrier composites with aligned graphene flakes and polyethylene molecules for high-pressure gas storage tanks. J. Energy Storage.

[46] Widakdo J., Huang T.J., Subrahmanya T.M., Austria H.F., Chou H.J., Hung W.S., Wang C.F., Hu C.C., Lee K.R., Lai J.Y. (2022). Bioinspired ionic liquid-graphene based smart membranes with electrical tunable channels for gas separation. Appl. Mater. Today.

[47] Widakdo J., Huang T.H., Subrahmanya T.M., Austria H.F., Hung W.S., Wang C.F., Hu C.C., Lee K.R., Lai J.Y. (2021). Tailoring of grapheneeorganic frameworks membrane to enable reversed electrical-switchable permselectivity in CO_2_ separation. Carbon.

[48] Liu L., Liu X., Liu Z., Zhang S.J., Qian L.B., Chen Z.Y., Li J.J., Fang P.F., He C.Q. (2022). High-performance fuel cells using Nafion composite membranes with alignment of sulfonated graphene oxides induced by a strong magnetic field. J. Membr. Sci..

[49] Hareesh K., Rondiya S.R., Dzade N.Y., Dhole S.D., Williams J., Sergey S. (2021). Polymer-wrapped reduced graphene oxide/nickel cobalt ferrite nanocomposites as tertiary hybrid supercapacitors: Insights from experiment and simulation. J. Sci. Adv. Mater. Dev..

[50] Wu J.R., Huang G.S., Pan Q.Y., Zheng J., Zhu Y.C., Wang B. (2007). An investigation on the molecular mobility through the glass transition of chlorinated butyl rubber. Polymer.

[51] Han X.B., Gao J., Chen Z.Y., Tang X.Q., Zhao Y., Chen T. (2020). Correlation between microstructure and properties of graphene oxide/waterborne polyurethane composites investigated by positron annihilation spectroscopy. RSC Adv..

[52] Han X.B., Chen T., Zhao Y., Gao J., Sang Y.N., Xiong H.H., Chen Z.Y. (2021). Graphene/polyethylene composites investigated by positron annihilation lifetime spectroscopy. Nanomaterials.

[53] He C.Z., She X.D., Peng Z., Zhong J.P., Liao S.Q., Gong W., Liao J.H., Kong L.X. (2015). Graphene networks and their influence on free-volume properties of graphene-epoxidized natural rubber composites with a segregated structure: Rheological and positron annihilation studies. Phys. Chem. Chem. Phys..

[54] Hebbar V., Bhajantri R.F., Ravikumar H.B., Ningaraju S. (2019). Role of free volumes in conducting properties of GO and rGO flled PVAPEDOT:PSS composite free standing flms: A positron annihilation lifetime study. J. Phys. Chem. Solids.

[55] Zhong J., Gao S., Xue G.B., Wang B. (2015). Study on Enhancement mechanism of conductivity induced by graphene oxide for polypyrrole nanocomposites. Macromolecules.

[56] Diwan P., Harms S., Raetzke K., Chandra A. (2012). Polymer electrolyte-graphene composites: Conductivity peaks and reasons thereof. Solid State Ionics.

[57] Mohsen M., Ashry A., Ismail A.M., Sayed F.E., Maqsoud D.M., Mahmoud K.R. (2019). Comparative effect of gamma irradiation on the nano-free volume and electrical properties of PVA/PEG/reduced graphene oxide nanocomposites. Arab J. Nucl. Sci. Appl..

[58] Zhong J., Ding Y., Gao F., Wen J., Zhou J.Y., Zheng W.B., Shen L., Fu C.Q., Wang B. (2019). Free volume correlation with electrical conductivity of polycarbonate/reduced graphene oxide nanocomposites studied by positron annihilation lifetime spectroscopy. J. Appl. Polym. Sci..

[59] He G.W., Huang S.Q., Villalobos L., Vahdat M.T., Guiver M.D., Zhao J., Lee W.C., Mensi M., Agrawal K.V. (2020). Synergistic CO2-sieving from polymer with intrinsic microporosity masking nanoporous single-layer graphene. Adv. Funct. Mater..

[60] Lecaros R.L., Deseo K.M., Hung W.S., Tayo L.L., Hu C.C., An Q.F., Tsai H.A., Lee K.R., Lao J.Y. (2019). Influence of integrating graphene oxide quantum dots on the fine structure characterization and alcohol dehydration performance of pervaporation composite membrane. J. Membr. Sci..

[61] Fan J.J., Zhou W., Wang Q., Chu Z.J., Yang L.Q., Yang L., Sun J., Zhao L., Xu J.M., Liang Y.J. (2018). Structure dependence of water vapor permeation in polymer nanocomposite membranes investigated by positron annihilation lifetime spectroscopy. J. Membr. Sci..

[62] Gaska K., Kadar R., Rybak A., Siwek A., Gubanski S. (2017). Gas barrier, thermal, mechanical and rheological properties of highly aligned graphene-LDPE nanocomposites. Polymers.

[63] Lecaros R.L., Bismonte M.E., Doma B.T., Doma B.T., Hung W.S., Hu C.C., Tsai H.A., Huang S.H., Lee K.R., Lai J.Y. (2020). Alcohol dehydration performance of pervaporation composite membranes with reduced graphene oxide and graphene quantum dots homostructured filler. Carbon.

[64] Xue G.B., Zhong J., Gao S., Wang B. (2016). Correlation between the free volume and thermal conductivity of porous poly(vinyl alcohol)/reduced graphene oxide composites studied by positron spectroscopy. Carbon.

[65] Madzarevic Z., Schut H., Cizek J., Dingemans T.J. (2018). Free volume in poly(ether imide) membranes measured by positron annihilation lifetime spectroscopy and Doppler broadening of annihilation radiation. Macromolecules.

[66] Biganeh A., Kakuee O., Kheiri H.R., Rachti M.L., Sheikh N., Yahaghi E. (2020). Positron annihilation lifetime and Doppler broadening spectroscopy of polymers. Radiat. Phys. Chem..

[67] Liang F., Liu D.X., Dong S.R., Zhao J., Cao X.Z., Jin W.Q. (2022). Facile construction of polyzwitterion membrane via assembly of graphene oxide-based core-brush nanosheet for high-efficiency water permeation. J. Membr. Sci..

[68] Lee J.Y., Zhan J.Y., Ang M.B., Yeh S.C., Tsai H.A., Jeng R.J. (2021). Improved performance of nanocomposite polyimide membranes for pervaporation fabricated by embedding spirobisindane structure-functionalized graphene oxide. Sep. Purif. Technol..

[69] Chang C.M., Chen H.T., Chuang S.H., Tsai H.C., Hung W.S., Lai J.Y. (2021). Mechanisms of one-dimensional and two-dimensional synergistic thermal responses on graphene oxide-modified PNIPAm framework membranes for control of molecular separation. Sep. Purif. Technol..

